# Unusual sequence characteristics of human chromosome 19 are conserved across 11 nonhuman primates

**DOI:** 10.1186/s12862-020-1595-9

**Published:** 2020-02-27

**Authors:** R. Alan Harris, Muthuswamy Raveendran, Kim C. Worley, Jeffrey Rogers

**Affiliations:** 1 Human Genome Sequencing Center, Baylor College of Medicine, One Baylor Plaza, Mail Stop: BCM226, Houston, TX 77030 USA; 2 Department of Molecular and Human Genetics, Baylor College of Medicine, One Baylor Plaza, Mail Stop: BCM226, Houston, TX 77030 USA

**Keywords:** Chromosome evolution, GC content, CpG sites, Primates, Variants, Gene regulatory elements

## Abstract

**Background:**

Human chromosome 19 has many unique characteristics including gene density more than double the genome-wide average and 20 large tandemly clustered gene families. It also has the highest GC content of any chromosome, especially outside gene clusters. The high GC content and concomitant high content of hypermutable CpG sites raises the possibility chromosome 19 exhibits higher levels of nucleotide diversity both within and between species, and may possess greater variation in DNA methylation that regulates gene expression.

**Results:**

We examined GC and CpG content of chromosome 19 orthologs across representatives of the primate order. In all 12 primate species with suitable genome assemblies, chromosome 19 orthologs have the highest GC content of any chromosome. CpG dinucleotides and CpG islands are also more prevalent in chromosome 19 orthologs than other chromosomes. GC and CpG content are generally higher outside the gene clusters. Intra-species variation based on SNPs in human common dbSNP, rhesus, crab eating macaque, baboon and marmoset datasets is most prevalent on chromosome 19 and its orthologs. Inter-species comparisons based on phyloP conservation show accelerated nucleotide evolution for chromosome 19 promoter flanking and enhancer regions. These same regulatory regions show the highest CpG density of any chromosome suggesting they possess considerable methylome regulatory potential.

**Conclusions:**

The pattern of high GC and CpG content in chromosome 19 orthologs, particularly outside gene clusters, is present from human to mouse lemur representing 74 million years of primate evolution. Much CpG variation exists both within and between primate species with a portion of this variation occurring in regulatory regions.

## Background

The unusual nature of human chromosome 19 has been noted since before the publication of the initial paper describing it’s DNA sequence [1]. One unusual aspect of human chromosome 19 is a gene density more than double the genome-wide average including 20 large tandemly clustered gene families [1]. Concomitant with the tandemly clustered gene families, chromosome 19 also contains a large number of segmental duplications with 6.2% of the sequence lying within intrachromosomal segmental duplications [1]. Sequence divergence between intrachromosomal segmental duplications suggests that many of the duplications occurred between 30 and 40 million years ago (MYA). This falls within the time range proposed for the anthropoid primate radiation with 40 MYA being close to the proposed Old World monkey/New World monkey divergence time of 43 MYA [2]. These duplication events could have implications for the evolution of phenotypic traits influenced by genes present on chromosome 19 across primates including human. Chromosome 19 also has an unusually high repeat content of 55%, consisting largely of Alu repeats, which comprise 26% of the chromosome [1].

One striking aspect of chromosome 19 is that it has the highest GC content (48%) of any human chromosome. The genome wide average GC content is 41%. This provides an opportunity for extensive gene regulation through DNA methylation at CpG sites in promoters, CpG islands and enhancers. CpG sites are hypermutable due to spontaneous deamination of methylated cytosines to form thymines. C to T changes at CpG sites show a higher substitution rate compared to non-CpG sites [3] and therefore one might expect higher than average rates of sequence changes on this chromosome.

The potential hypermutability of the large number of chromosome 19 CpG sites together with its high gene density raises the possibility that chromosome 19 may exhibit a large amount of intra- and inter-species variation in DNA sequence and methylation regulation arising from single nucleotide polymorphisms (SNP) or fixed base substitutions, respectively, that disrupt CpG sites. This is particularly interesting in the context of primate evolution given the long standing hypothesis, first proposed by King and Wilson [4], that “The organismal differences between chimpanzees and humans would then result chiefly from genetic changes in a few regulatory systems, while amino acid substitutions in general would rarely be a key factor in major adaptive shifts.” This concept has been extended beyond human-chimpanzee comparisons to encompass primate evolution in general [5, 6]. Likewise, intra-species variants affecting gene regulation are the differences upon which positive selection can act and conversely may identify regulatory variants that cause dysfunction involved in disease processes unrelated to amino acid changes. The combination of unusual GC content together with potential regulatory variation that may arise from chromosome 19 hypermutability make this chromosome a prime candidate for evolutionary genomic analyses.

## Results

### GC content and CpG density

**Fig. 1:**
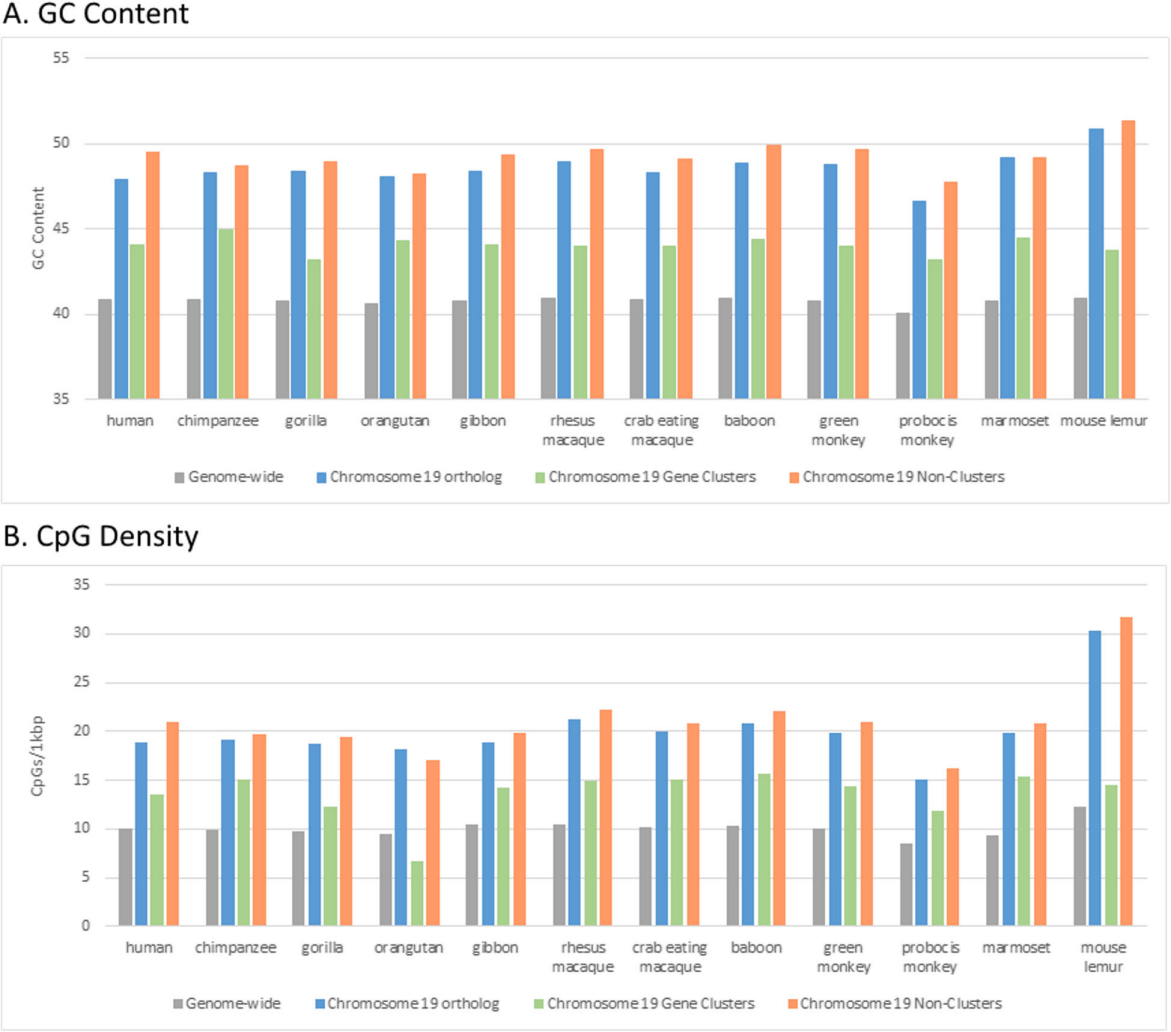
GC content **a** and CpG density **b** of human chromosome 19 and nonhuman primate orthologs. GC content and CpG density partitioned into the gene cluster and non-cluster regions of the chromosome 19 are also shown

In order to establish the patterns of chromosomal GC content across humans and nonhuman primates (NHP), we identified the orthologs of human chromosome 19 in 11 NHP genome assemblies that have scaffolds assigned to chromosomes (Table S1). We then compared characteristics of the chromosome 19 orthologs to other autosomes and the X chromosome. The Y chromosome was not examined because it is only available for 3 of the NHP assemblies. Most primate genome assemblies include a single chromosome that is orthologous to human chromosome 19, but there are exceptions. In the mouse lemur (*Microcebus murinus*), which diverged from the lineage leading to humans around 74 MYA [2], the orthologous chromosomes are MIM22 and MIM24. The ancestral haplorhine primate experienced a fusion of 19p and 19q relative to strepsirhine primates [7]. In the highly rearranged gibbon (*Nomascus leucogenys*) genome [8] the orthologous chromosomes are parts of NLE10, NLE11 and NLE17. In this study, we calculated GC content by chromosome or, in the case of gibbon, chromosome 19 orthologous segments [9, 10] (Table S2). The NHP orthologs of human chromosome 19 display higher GC content than any other chromosome for all species examined. The average GC content of chromosome 19 orthologs is 48.55%, ranging from a high of 50.84% in mouse lemur to a low of 46.64% in the proboscis monkey (*Nasalis larvatus*) (Fig. 1a, Table S2, Fig. S1). Across these 12 species, the average GC content genome-wide is 40.78% ranging from 40.96% in rhesus to 40.05% in proboscis monkey. Furthermore, GC content shows a significant (*p* < 0.05) negative correlation with chromosome length in 8 of the 12 primate genomes we examined (Table S3).

To extend the phylogenetic breadth of these comparisons, we analyzed GC content in mouse, dog and three NHP genomes with scaffold assemblies only (squirrel monkey (*Saimiri boliviensis*), tarsier (*Tarsius syrichta*), and bushbaby (*Otolemur garnettii*)). We used the UCSC chains and liftOver software to reciprocally lift over the orthologous segments of human chromosome 19 [11]. Liftover was performed at minMatch parameter settings ranging from 0.1 to 1 (Table S4). In tarsier the chromosome 19 orthologous sequences exhibited the highest GC content of all chromosomes, at all liftOver settings. However, for squirrel monkey and bushbaby, orthologs of chromosome 19 showed the highest GC content at some liftOver settings, while chromosome 22 orthologs showed higher GC content at other settings. The chromosome 19 ortholog in the dog genome showed the highest GC content at lower liftOver stringency, but chromosome 22 has higher GC content at more stringent liftOver settings. Mouse showed highest GC content for chromosomes 19, 22, 16, or 17 depending on the liftOver settings used. It has been suggested that mouse orthologs of human chromosome 19 do not show particularly high GC content based on syntenic blocks [1] and gene orthologs [12]. Taken together, these results show that across a wide series of mammals, and especially among anthropoid primates, the orthologs of human chromosome 19 exhibit relatively high GC content. In most species we examined, the GC content is higher in the chromosome 19 ortholog than any other chromosome.

**Fig. 2:**
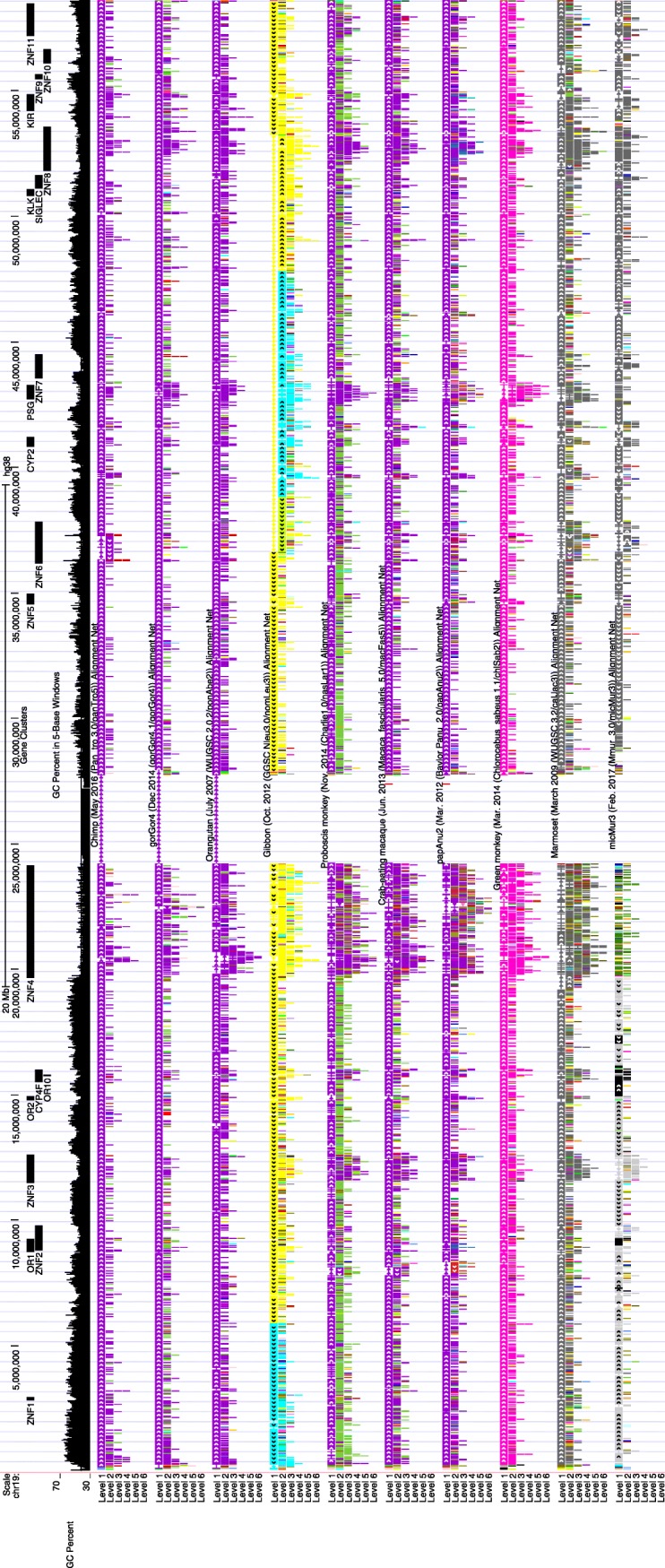
UCSC Browser view of primate Alignment Nets on human (hg38) chromosome 19. GC percent and the locations of the 20 tandem gene clusters are also shown

We next examined GC content in the context of Ensembl gene annotations [13] for 11 of the primates excluding proboscis monkey which has not been annotated. Gene density in 100kbp windows averaged by chromosome shows a positive correlation with chromosome GC content (Fig. S2). In species where the chromosome 19 ortholog is a single chromosome, the chromosome lies at the upper range for both gene density and GC content. We also compared GC content in genic and intergenic regions based on Ensembl gene annotations. Chromosome 19 genic regions in each species examined consistently had higher GC content (50.62% average, see Table S2 for individual species) compared to intergenic regions (46.61% average, see Table S2 for individual species) across the primates. Genic GC content ranged from a high of 52.77% in mouse lemur to a low of 49.09% in human. To further examine GC content in the context of genes, we partitioned human chromosome 19 into gene cluster regions consisting of 20 previously identified clusters [1] and contrasted those clusters with regions outside of the clusters (Fig. 2). The human clusters have a GC content of 44.03% which is slightly higher than the previously reported 43.1% [1]. The regions outside of the clusters had a GC content of 49.51% which is slightly lower than the 50.3% GC content reported for regions of clear 1:1 human/mouse orthology [1].

The human clusters were lifted over to the NHP genomes. Due to the repetitive nature of the cluster regions, liftOver from human had to be performed allowing for multiple mappings. Only mappings to the chromosome 19 orthologous chromosomes were retained. The lifted over regions were then merged if within 100kbp of each other and only merged regions with a minimum length of 80kbp were retained based on the smallest human cluster region being 87,645 bp in length. The pattern of higher GC content in regions outside of the gene clusters compared to the gene clusters was consistent across the primates including mouse lemur (Table S5).

Moving beyond GC content, we next examined the density of CpG sites and CpG islands [9, 14] by chromosome in primates (Fig. 1b, Table S2). Chromosome 19 orthologs consistently showed the highest density of CpG sites (average 20.11 sites/kbp) across all the primates. The ratio of observed CpG sites to expected CpG sites based on GC content was 0.34 averaged across chromosome 19 orthologs compared to 0.25 for other chromosomes. Chromosome 19 ortholog CpG observed/expected ratios ranged from a high of 0.47 in mouse lemur to a low of 0.28 in the proboscis monkey. CpG islands were also most prevalent on chromosome 19 orthologs whether looking at all sequence (48.53 CpG island bases/kbp) or only repeat masked sequence (35.34 CpG island bases/kbp). In relation to the gene clusters, CpG sites and CpG islands both showed a greater abundance outside of the gene clusters, which is consistent with the patterns seen for GC content (Table S5).

We also examined CpG density in the context of Ensembl regulatory features [15] annotated on the human genome consisting of promoter, promoter flanking and enhancer regions. Chromosome 19 has the greatest density of CpGs in promoter flanking (29.39 CpGs/kbp of promoter flanking region) and enhancer (17.97 CpGs/kbp of enhancer region) regions (Table S6). The density of CpGs in regulatory features is higher outside of the gene clusters than in the gene clusters (Table S7) which is consistent with GC content.

### Intra-species variation

**Fig. 3:**
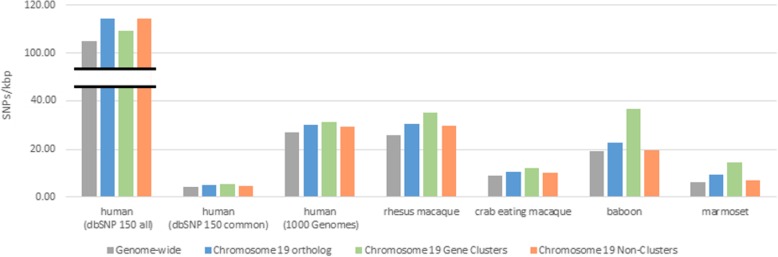
SNPs/kbp of human chromosome 19 dbSNP 150 all, dbSNP 150 common (MAF > 0.01), and 1000 Genomes datasets together with three Old World monkey orthologs and a New World monkey ortholog. SNPs partitioned into the gene cluster and non-cluster regions of the chromosome 19 are also shown

Intra-species variation provides the diversity upon which evolution can act, so we next examined the chromosomal distribution of single nucleotide polymorphisms (SNP) within species. For common human SNPs (MAF > = 0.01) in dbSNP 150 [16], chromosome 19 shows the highest density of SNPs (4.91/kbp) of any chromosome (Fig. 3, Table S8). The density of chromosome 19 SNPs in both all dbSNP records (common and rare variants) (114.19 SNPs/kbp) and 1000 Genomes data [17] (30.08 SNPs/kbp) is the third highest of all chromosomes. While chromosome 19 does not show the highest density in these latter two datasets, the density is greater than the genome wide average density for both all dbSNP (104.89 SNPs/kbp) and 1000 Genomes data (27.27 SNPs/kbp). When human chromosome 19 is partitioned into gene cluster and non-cluster regions, the non-cluster regions show greater SNP density for all dbSNP SNPs. However, the cluster regions show greater density for common dbSNP and 1000 Genome variants (Table S9). The pattern of more sequence variants in non-cluster regions seen in all dbSNP variants could be attributable to lower quality variant calls in this largely unfiltered dataset.

We next examined SNPs in three Old World monkeys and one New World monkey for which adequate data are available. For rhesus macaque (*Macaca mulatta*) we used our catalog of rhesus variants [18] which has now been expanded to include 526 individuals. For the crab eating macaque (*Macaca fascicularis*) we examined SNPs from 26 individuals [19]. We also examined 15 baboons (genus *Papio*) representing 6 baboon species plus one sample from the related gelada (genus *Theropithecus)* [20]. For a New World monkey representative we used common marmoset (*Callithrix jacchus*) SNPs from 9 individuals [21]. The chromosome 19 orthologs showed the highest density of SNPs among all chromosomes for all of these monkeys (Fig. 3, Table S8). The SNP density in gene cluster regions is higher than in non-cluster regions in the monkeys (Table S9) which is the pattern that was seen for human common dbSNP and 1000 genome variants but not for all dbSNP variants.

In order to assess any potential effect of CpG hypermutability on SNPs we identified SNPs occurring in CpG dinucleotides. Human chromosome 19 shows the highest percentage of SNPs in CpG dinucleotides (SNPs in CpG / total SNPs) when looking at all dbSNPs (18.56%) or 1000 Genomes SNPs (24.85%) and second highest percentage when looking at common dbSNPs (22.74%) (Table S8). The same pattern of chromosome 19 orthologs showing the highest percentage of CpG SNPs is observed in all the nonhuman primates examined (Table S8).

We examined potential functional consequences of SNPs on gene expression levels across individuals using Genotype-Tissue Expression (GTEx) [22] RNA-Seq data in the form of a Transcripts Per Million (TPM) expression matrix. GTEx samples from the same seven tissues (brain, heart, kidney, liver, lung, skeletal muscle and testis) that were examined for expression across mammals by Chen et al. [23] (see below) were identified and the median variance by chromosome was calculated (Table S10). The median expression variance of chromosome 19 was greater than all other chromosomes across all seven tissues. Chromosome 19 also showed the highest expression levels based on TPM averages. TPM values were averaged across individuals for each gene. The averaged gene TPM values were averaged across chromosomes separately in brain, kidney, lung and skeletal muscle (Table S11). We calculated the index of dispersion as the ratio of TPM expression variance to TPM expression average for each chromosome (Table S12). Chromosome 19 has the highest index of dispersion in all tissues with the exception of skeletal muscle in which it has the second highest index of dispersion. The high index of dispersion of chromosome 19 compared to the other chromosomes indicates that it has a higher degree of expression variability.

### Inter-species variation

Evolutionary action on the intra-species variation we describe above results in inter-species fixed differences and, alternatively, sequence conservation. We explored evolutionary signatures across three sets of vertebrate genomes through the use of phyloP [24] scores generated from multiple sequence alignments to the human hg38 genome assembly. The 100 vertebrate species (phyloP100way, 11 NHP), 20 mammalian species (phyloP20way, 16 NHP, tree shrew, mouse, dog), and 7 mammalian species (phyloP7way, human, chimpanzee, rhesus, mouse, rat, dog, opossum) phyloP datasets were used. phyloP scores measure conservation at single nucleotides and also identify accelerated nucleotide evolution, represented as a negative phyloP score, which may arise from positive selection.

**Fig. 4:**
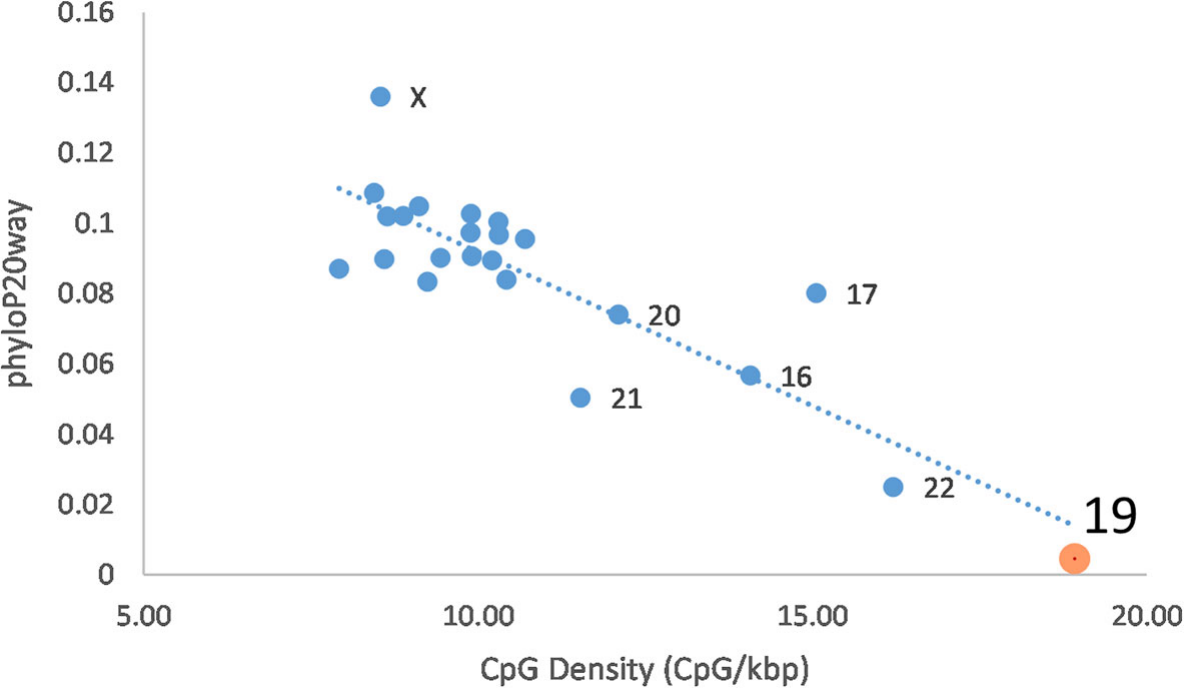
PhyloP20way scores compared to CpG density averaged by human chromosome. Chromosome 19 is highlighted

For phyloP scores calculated by chromosome, chromosome 19 is the least conserved, or most diverged, in the primate enriched 20 way dataset, the second most diverged chromosome in the 7 way dataset which contains 3 primates, and the third most diverged chromosome in the 100 way dataset (Table S13). CpG density compared to phyloP20way scores averaged by chromosome shows a significant negative Spearman’s correlation (r_s_ = − 0.7047; *p* = 0.000175) with chromosome 19 being at the extreme in both CpG density and phyloP20way score (Fig. 4). PhyloP scores by chromosome were calculated for CpG islands and Ensembl regulatory features consisting of promoter, promoter flanking and enhancer regions [15] (Table S14). Chromosome 19 promoters were the second least conserved in all phyloP datasets. Promoter flanking regions were the least conserved, second least conserved, or third least conserved in the phyloP20way, phyloP7way, and phyloP100way respectively. Enhancers were the least conserved in the phyloP20way and phyloP7way datasets and the second least conserved in the phyloP100way. Chromosome 19 phyloP100way scores showed accelerated evolution for promoter flanking (− 0.0064) and enhancer (− 0.026) regions (Table S14). These regulatory regions were also identified as having the highest CpG content in human chromosome 19 compared to all other chromosomes (Table S6). For phyloP100way there are signals of acceleration for enhancers in both the chromosome 19 gene cluster (− 0.048) and non-cluster (− 0.025) regions (Table S15).

Individual regulatory features showing signs of acceleration were partitioned into gene cluster and non-cluster regions and analyzed using the Genomic Regions Enrichment of Annotations Tool (GREAT) [25]. GREAT assesses the functional significance of cis-regulatory regions by examining both proximal and distal genes and associated annotations from 20 ontologies. GREAT controls for false positives using a binomial test over the input genomic regions. For the gene cluster regions, enhancers showing acceleration based on all phyloP datasets had the Gene Ontology (GO) Biological Process term “female pregnancy” ranked as number one based on Binomial and Hypergeometric Rank (Table S16). The genes associated with this term were in the pregnancy-specific glycoproteins (PSG) cluster. PSG genes have immunoregulatory, pro-angiogenic, and anti-platelet functions and low levels of PSG are associated with pregnancy pathologies [26]. In promoter flanking regions the highest ranking GO Molecular Function and Biological Process terms were ones related to immune response composed of the Killer Cell Immunoglobulin Like Receptor (KIR) and Leukocyte Immunoglobulin Like receptor (LILR) genes. Promoters showed enrichment for numerous terms related to zinc finger transcription factor binding across all phyloP datasets.

**Fig. 5:**
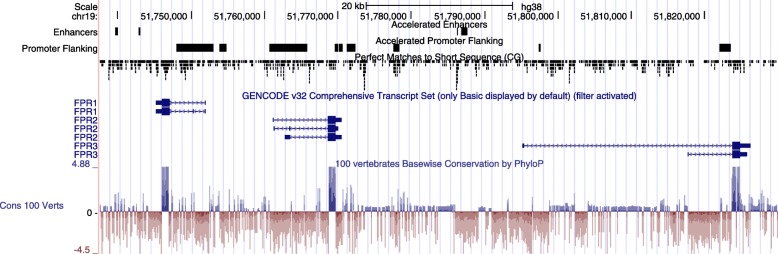
UCSC Browser view of the human formyl peptide receptor region showing enhancer and promoter flanking regulatory regions showing accelerated nucleotide evolution and phyloP scores. The location of CpGs are also shown

For the non-cluster regions, the GO term “N-formyl peptide receptor activity”, which is involved in mediating immune cell response to infection, was the number one ranking Molecular Function term, based on Hypergeometric Rank, for enhancers in all phyloP datasets, as well as for promoter flanking regions based on phyloP100way and phyloP20way datasets (Fig. 5, Table S17). N-formyl peptide receptor reached significance in the enhancer phyloP100way (HyperFdrQ = 0.0011) and phyloP20way (HyperFdrQ = 0.015) and promoter flanking phyloP100way (HyperFdrQ = 0.00026). Genes associated with “N-formyl peptide receptor activity” are Formyl Peptide Receptor 1, 2 and 3 (*FPR1*, *FPR2*, *FPR3*) and Complement C5a Receptor 1 and 2 (*C5AR1* and *C5AR2*).

In order to assess the effect of CpG hypermutability on substitutions we identified substitutions based on Ensembl EPO multiple alignments of primates [27, 28] and partitioned the substitutions into those in CpG dinucleotides and those occurring in other contexts. The percent of substitutions occurring in CpGs in pairwise comparisons to human chromosomes is shown in Table S18. In human pairwise comparisons to each Old World monkey, marmoset and mouse lemur chromosome 19 has the highest percent of substitutions in CpGs. In human pairwise comparisons to great apes and gibbon chromosome 19 has the second highest percent of substitutions in CpGs after chromosome 22.

We examined potential functional consequences of nucleotide diversity across species on gene expression levels using previously published RNA-Seq data. Chen et al. [23] examined RNA-Seq across seven different tissues from 17 mammalian species, including human (*n* = 1–6 individuals depending on tissue), great apes (*n* = 2–13 individuals depending on tissue), rhesus macaque (*n* = 1–6 individuals depending on tissue) and marmoset (*n* = 0–2 individuals depending on tissue). They calculated expression variance separately for each individual tissue across species for each of 10,899 one-to-one orthologs. We calculated the average variance by human chromosome (Table S19) and chromosome 19 shows the most variance across species in heart and skeletal muscle, the second most variance across species in brain, liver and lung, and the third most variance across species in kidney. In testis, chromosome 19 shows the sixth most variance with chromosome X showing the most variance.

### Primate sperm Methylome data

Germline DNA methylation can potentially leave an evolutionary signature in the genome due to the hypermutability of methylated cytosines and less mutable nature of unmethylated cytosines. A comparison of sperm methylome data across human, chimpanzee, gorilla, rhesus, mouse, rat and dog found a genome wide evolutionary expansion of hypomethylation at CpG sites [29]. Primates and dog show a pattern of widening hypomethylation around transcription start sites into promoter flanking regions while rodents evolve new hypomethylated regions. We compared methylation levels of promoter, promoter flanking, enhancer and general genomic regions between primate chromosome 19 orthologs and whole genomes using the methylation data from Qu, et al. [29]. The promoter flanking and general genomic regions showed hypomethylation of chromosome 19 relative to genome wide levels in all the examined primates consisting of human, chimpanzee, gorilla and rhesus (Table S20), with an average methylation level 0.09 lower, while promoter and enhancer methylation was similar between chromosome 19 and the genome wide levels in all the examined primates consisting of human, chimpanzee, gorilla and rhesus (Table S20). Extensive hypomethylation of chromosome 19 promoter flanking regions may represent an extreme case of the primate pattern of expanding hypomethylation around promoters. In all the examined primates consisting of human, chimpanzee, gorilla and rhesus the average size of hypomethylated regions (HMRs) [29] were longer on chromosome 19 than the genome wide average by an average across species of 222 bp (see Table S21 for HMR lengths by species). Furthermore, the average size of Ensembl promoter flanking regions on human chromosome 19 (1519 bp) is 207 bp longer than the genome wide average (1311 bp). The chromosome wide hypomethylation of primate chromosome 19 may be due to selection to maintain methylation levels lower than the genome wide average.

## Discussion

The high GC content and CpG density of human chromosome 19 is evolutionarily conserved across nonhuman primate orthologs ranging from 1-to-1 chromosome orthologs in the haplorhine great apes and monkeys to the two orthologous chromosomes in the strepsirhine mouse lemur. This conservation even extends to the gibbon genome which has been heavily rearranged compared to other primates [8]. Although human chromosome 19 is orthologous to four distinct gibbon chromosome segments, those segments nevertheless display the same elevated GC and CpG levels as other nonhuman primate chromosome 19 orthologs. Despite these rearrangements, genome wide conservation of gibbon topologically associating domains (TADs), functional compartments in which intra-genomic interactions occur, along with their epigenomic states, including DNA methylation, has been reported [30]. This is consistent with our finding that the gibbon orthologs of the CpG rich chromosome 19 remain CpG rich and presumably epigenomically conserved despite rearrangements.

Chromosome size negatively correlates with recombination rates with smaller chromosomes having higher recombination rates per Mbp than larger chromosomes [31]. GC biased gene conversion arises from recombination thus GC content is expected to be higher in shorter chromosomes undergoing higher recombination rates and this pattern has been observed in a number of eukaryotes [31]. We found a significant (*p* < 0.05) negative correlation between chromosome length and GC content in 8 of the 12 primate genomes we examined (Table S3). Gibbon is one of the genomes that does not show this correlation possibly due to its heavily rearranged nature in which chromosome lengths evolve rapidly. Among the primates in which chromosome 19 is a single chromosome the average length of the chromosome is 56,156,097 bp and the average GC content is 48.34%. Mouse lemur chromosomes 22 (30,143,674 bp; 49.34% GC) and 24 (15,088,824 bp; 53.63% GC) are both smaller in size and higher in GC content than the chromosome 19 orthologs in the other primates. It is possible that the fusion of 19p and 19q in haplorhine primates relative to strepsirhine primates started a trend toward decreasing GC content due to an increase in chromosome size.

The conservation of high GC content and CpG density across human chromosome 19 orthologs in spite of the hypermutability of CpG sites, the high intraspecies variation and the greater interspecies divergence at the nucleotide level seems contradictory. It is not obvious how the high GC and CpG levels have been retained across multiple branches of the primate phylogeny in the face of significantly elevated rates of sequence change (low sequence conservation). Our analyses demonstrate that despite the high turnover of sequence on chromosome 19 orthologs, there is consistent maintenance of unusually high GC and CpG levels across multiple species. It is possible that the high GC content and CpG density of chromosome 19 orthologs is a remnant of the even higher GC content of the ancestral smaller chromosomes that fused to form haplorhine chromosome 19 and are still present in mouse lemur. The somewhat lower GC content seen in humans, apes and monkeys could be an intermediate stage before nucleotide changes have had enough time to lower chromosome 19 GC content to be more in line with its size.

SNPs provide diversity upon which evolution can act. Two human SNP datasets together with SNPs identified in three Old World monkey species and a New World monkey species all show higher SNP density for the chromosome 19 orthologs than the genome-wide average. Common (MAF > = 0.01) dbSNP human SNPs and all the nonhuman primate SNPs rank chromosome 19 orthologs as highest in SNP density. Furthermore, the proportion of SNPs that lie in CpG sites are highest for the human datasets, except for common dbSNP, and for all of the nonhuman SNP datasets. In summary, chromosome 19 orthologs show an exceptionally large amount of intraspecific SNP diversity across primates ranging from human to a New World monkey and much of this diversity is in CpG sites.

Human chromosome 19 shows the highest CpG density of any chromosome in promoter flanking and enhancer regions. These same enhancer and promoter flanking regions also show the greatest amount of divergence in the primate enriched phyloP20way dataset and accelerated nucleotide evolution in the phyloP100way dataset. Individual enhancer and promoter flanking regions showing accelerated nucleotide evolution are associated with genes enriched for immune or pregnancy related GO terms. The genes associated with the pregnancy GO term are pregnancy-specific glycoproteins (PSG) which also have an immunoregulatory function [26]. Immunity and reproduction related genes are frequently identified as undergoing positive selection in genome-wide scans for selection based on protein coding changes [32]. Selection could also be acting on the regulatory potential of the enhancer and promoter flanking regions, thereby influencing associated immune or reproductive functions.

The GO term “N-formyl peptide receptor activity” is the highest ranking term for enhancer and promoter flanking regions that show accelerated nucleotide evolution. This provides one clear example of accelerated evolution involving immunity related genes. Formyl peptide receptors are involved in mediating immune cell response to infection. Phylogenetic analysis [33] showed that an early duplication generated *FPR1* and *FPR2*/*FPR3* with *FPR3* arising from a later duplication near the origin of primates. FPR1 and FPR2 show evidence of positive selection at sites located in the extracellular loops of the protein, while selective pressures may be relaxed on FPR3. It has been suggested that positive selection of mammalian FPRs links nucleotide changes to changes in the surface structure of the protein that is important for defense against pathogens [33]. The same selective forces acting on the protein sequences of *FPR1* and *FPR2* could also be acting on their associated enhancer and promoter flanking regions resulting in accelerated nucleotide evolution.

Another example of activity that protects cells from biological agents is the transcriptional silencing of endogenous retroviruses (ERVs). Krueppel-Associated Box (KRAB)-associated protein 1 (KAP1) epigenetically represses endogenous retroviral DNA through targeting by KRAB-containing zinc finger transcription factors (TFs) [34]. Many of these zinc finger TFs exist in clusters on chromosome 19 that are hotspots for copy number variation [34]. During primate evolution, zinc finger TFs arise for each ERV family that enters the genome and the zinc finger TFs are preferentially located on chromosome 19 [34]. In our GO analysis of accelerated regulatory regions, chromosome 19 promoters showed enrichment for numerous terms related to zinc finger transcription factor binding across all phyloP datasets. The same evolutionary forces driving zinc finger TF copy number variation in response to ERV invasion could also be acting on zinc finger TF promoters resulting in accelerated nucleotide evolution.

The high CpG content of chromosome 19 orthologs has implications for their DNA methylation regulatory potential. The regulatory portion of the human methylome has been identified based on comparisons of methylation levels across multiple tissues [35]. If chromosomes are ranked based on the proportion identified as regulatory methylome, chromosome 19 has the third greatest amount of regulatory potential (6.33% of the chromosome length) after chromosomes 22 and 17 (7.71 and 6.76% respectively). The high GC content chromosome 19 regions lying outside of the gene clusters consist of 7.43% regulatory methylome. It is reasonable to think that similar patterns of methylation regulatory potential are present in chromosome 19 nonhuman primate orthologs with high CpG content. Therefore, the retention of high CpG levels may be related to conservation of regulatory sites. Assays measuring regulatory activity of candidate hominoid-specific liver enhancer orthologs across 11 primates reveals the evolutionary-functional trajectories of the enhancers [36]. Nucleotide differences that correlated with functional changes are enriched for cytosine deamination events in CpGs.

From this study, we learn for the first time that chromosome level sequence features such as GC content and CpG density are conserved over millions of years of primate evolutionary change, despite the substantially higher rate of mutation in CpG dinucleotides. Our findings provide the empirical justification for future studies that explore potential mechanisms including negative selection or GC biased gene conversion in short chromosomes acting to conserve those genomic features. Our findings can also serve to stimulate similar analyses of other mammalian clades. Do similar patterns of conservation of GC content, CpG density, SNP frequency and related features characterize specific chromosomes within the carnivores, the bats or the artiodactyls? Does the conservation and dynamics observed here for the orthologs of human chromosome 19 across primate phylogeny extend to other mammalian groups, or is it a different chromosome that maintains extreme GC content and CpG density? Is the relationship between high CpG content and accelerated nucleotide evolution in promoter flanking and enhancer regulatory elements seen in nonprimate species? The large number of nonprimate species included in the phyloP100way dataset, which shows regulatory element accelerated nucleotide evolution, suggests it should be. These questions are beyond the scope of the present analysis, but our results suggest such analyses of other mammalian clades may reveal similar patterns. This work points to fundamental processes of genomic evolution that extend across lineages and deep time. Understanding the range of vertebrate clades in which similar correlations hold will provide greater insight into large-scale patterns of genomic conservation and change.

## Conclusions

We conclude that many of the features that make human chromosome 19 unusual among human chromosomes are shared across a wide range of primate orthologs. Gene content, GC content, CpG density and SNP density all appear higher in primate orthologs of human chromosome 19. This pattern illustrates that high CpG density and thus high regulatory potential has been conserved for tens of millions of years despite the hypermutability and accelerated nucleotide evolution observed across orthologs of human chromosome 19. While the chromosome wide pattern of CpG density is conserved, intra- and inter-species variability is present at individual CpG loci.

## Methods

### Genome assemblies and annotations analyses

Genome assembly fasta files, GC content as gc5Base.bw files, CpG island predictions, and liftOver chains for hg38, panTro5, gorGor4, ponAbe2, nomLeu3, rheMac8, macFas5, papAnu2, chlSab2, nasLar1 calJac3, micMur3, otoGar3, saiBol1, tarSyr2, mm10, and canFam3 were downloaded from the UCSC Genome Browser site [9]. Human (hg38) phyloP bigWig files and dbSNP 150 data were also downloaded from UCSC. Gene predictions and regulatory build were downloaded from the Ensembl site [13, 15]. EPO alignments [27, 28] across primates were downloaded from ftp://ftp.ensembl.org/pub/release-98/maf/ensembl-compara/multiple_alignments/13_primates.epo/. 1000 Genomes [17] vcf files lifted over to hg38 were downloaded from http://ftp.1000genomes.ebi.ac.uk/vol1/ftp/release/20130502/supporting/GRCh38_positions/. CpG sites were identified in assembly fasta files using in-house software. Bwtool [10] was used to calculate statistics from bigWig data for GC content and phyloP scores. Bedtools [37] was used to perform intersection and complementation operations among the datasets.

For primates with genome assemblies in which contigs and scaffolds are assigned to chromosomes, the chromosomes orthologous to HSA19 were used. For primate assemblies without chromosome assignments and the outgroups, reciprocal liftOver was performed in which the human chromosomes were lifted over to the nonhuman assembly and the resulting regions were lifted back over to human. Only segments which then lifted back to the original human coordinates were used.

### Nonhuman primate variant calling

Samples from rhesus macaque (*Macaca mulatta*) (*n* = 526), crab eating (or cynomolgus) macaque (*Macaca fascicularis*) (*n* = 26), olive baboon (*Papio anubis*) (*n* = 4), yellow baboon (*Papio cynocephalus*) (*n* = 2), guinea baboon (*Papio papio*) (*n* = 2), hamadryas baboon (*Papio hamadryas*) (*n* = 2), kinda baboon (*Papio kindae*) (*n* = 3), chacma baboon (*Papio ursinus*) (*n* = 2), and gelada (*Theropithecus gelada*) (*n* = 1) were analyzed for SNPs. Marmoset (*Callithrix jacchus*) (*n* = 9) SNP calls have been previously published [21].

BWA-MEM version 0.7.12-r1039 [38] was used to align the Illumina reads to the rhesus macaque (Mmul_8.0.1/rheMac8), crab eating macaque (Macaca_fascicularis_5.0/macFas5), or baboon (Panu2.0/papAnu2) reference assembly and generate BAM files. Picard MarkDuplicates version 1.105 (http://broadinstitute.github.io/picard/) was used to identify and mark duplicate reads. Variants were called using GATK version 3.3–0 following best practices for that version [39, 40]. HaplotypeCaller was used to generate gVCF files for each sample. Joint genotype calling was performed on all samples using GenotypeGVCFs to generate a VCF file. GATK hard filters (SNPs: “QD < 2.0 || FS > 60.0 || MQ < 40.0 || MQRankSum < -12.5 || ReadPosRankSum < -8.0”; Indels: “QD < 2.0 || FS > 200.0 || ReadPosRankSum < -20.0”) (https://software.broadinstitute.org/gatk/documentation/article?id=2806) were applied and calls that failed the filters were removed.

### RNA-Seq analysis

The Genotype-Tissue Expression (GTEx) version 8 RNA-Seq Transcripts Per Million (TPM) expression matrix was downloaded from https://storage.googleapis.com/gtex_analysis_v8/rna_seq_data/GTEx_Analysis_2017-06-05_v8_RNASeQCv1.1.9_gene_tpm.gct.gz. The calculated variance across mammals for seven tissues was downloaded from the EVolutionary Estimates of Expression (EVEE) Gene Browser here https://portals.broadinstitute.org/evee/static/data/evolutionary_statistics.zip.

## Supplementary information

**Additional file 1: Table S1.** Human chr19 orthologs. **Table S2.** Primate genome GC and CpG Content. **Table S3.** Spearman’s correlation coefficients of chromosome length to GC Content. **Table S4.** GC content for outgroups at varying liftOver settings. **Table S5.** GC content and CpG density for chromosome 19 orthologs partitioned into gene cluster and non-gene cluster regions. **Table S6.** Human Regulatory region CpG Density. **Table S7.** Human Regulatory region CpG Density partitioned into gene cluster and non-gene cluster regions. **Table S8.** SNPs in humans, three Old World monkeys and a New World monkey. **Table S9.** SNPs for chromosome 19 orthologs partitioned into gene cluster and non-gene cluster regions. **Table S10.** Human Expression Variance Based on GTEx. **Table S11.** Human Expression Transcripts Per Million (TPM) Based on GTEx. **Table S12.** Human Expression Index of Dispersion Based on GTEx. **Table S13.** PhyloP conservation relative to the human genome. **Table S14.** PhyloP conservation relative to the human genome for regulatory regions. **Table S15.** PhyloP conservation relative to the human genome for regulatory regions in chromosome 19 cluster and non-cluster regions. **Table S16.** GREAT results within gene clusters for the phyloP100way dataset for Enhancers, Promoter Flanking, and Promoters showing accelerated nucleotide evolution. **Table S17.** GREAT results outside of gene clusters for the phyloP100way dataset for Enhancers, Promoter Flanking, and Promoters showing accelerated nucleotide evolution. **Table S18.** Percent of substitutions in CpGs based on pairwise comparisons to human. **Table S19.** Mammalian Expression Variance. **Table S20.** Primate sperm methylome data 1 for promoter, promoter flanking, enhancer and general genomic regions. **Table S21.** Hypomethylated region average size genomewide and for chromosome 19.

**Additional file 2: Figure S1.** Nonhuman primate phylogenetic tree showing the chromosome 19 ortholog GC content for species in the tree. The tree topology is based on the species tree used in the Ensembl Compara pipelines (https://ensembl.org/info/genome/compara/species_trees.html). **Figure S2.** GC content compared to gene density in 100kbp windows of human and non-human primate chromosomes. A) Scatterplot of GC content compared to gene density by chromosome. Chromosome 19 orthologs are highlighted in red. B) Spearman’s correlation coefficients and *p* values for GC content compared to gene density.

## Data Availability

Genomes and associated annotations are available through UCSC (https://genome.ucsc.edu/) or Ensembl (http://www.ensembl.org). DOIs for nonhuman primate SNP calls in vcf format were registered through Zenodo. The nonhuman primate SNP call vcf files are available for download at the following URLs: rhesus macaque (https://zenodo.org/record/3515522), crab eating macaque (https://zenodo.org/record/3490984), baboon (https://zenodo.org/record/3515341), marmoset (https://zenodo.org/record/3490953). Nonhuman primate sequencing data used in this study are available through the following NCBI BioProject (https://www.ncbi.nlm.nih.gov/bioproject) accessions: rhesus macaque PRJNA251548, crab eating macaque PRJNA25734, baboon PRJNA260523, marmoset PRJNA20401.

## Abbreviations

bp: Base pairs
CpG: Cytosine phosphate Guanine
DNA: Deoxyribonucleic Acid
GC: Guanine Cytosine
GO: Gene Ontology
HMR: Hypomethylated Regions
kbp: Kilobase pairs
MAF: Minor Allele Frequency
MYA: Million Years Ago
NHP: Nonhuman Primate
SNP: Single Nucleotide Polymorphism
TAD: Topologically Associating Domains
TPM: Transcripts Per Million
