# Supplementary material for: Unusual sequence characteristics of human chromosome 19 are conserved across 11 nonhuman primates
Source: BMC Evol Biol. 2020 Feb 27;20:33. doi: 10.1186/s12862-020-1595-9 (PMC7045612; doi:10.1186/s12862-020-1595-9)
Supplement: Supplementary file 2 — Additional file 2: Figure S1. Nonhuman primate phylogenetic tree showing the chromosome 19 ortholog GC content for species in the tree. The tree topology is based on the species tree used in the Ensembl Compara pipelines (https://ensembl.org/info/genome/compara/species_trees.html). Figure S2. GC content compared to gene density in 100kbp windows of human and non-human primate chromosomes. A) Scatterplot of GC content compared to gene density by chromosome. Chromosome 19 orthologs are highlighted in red. B) Spearman’s correlation coefficients and p values for GC content compared to gene density. [file 12862_2020_1595_MOESM2_ESM.docx]

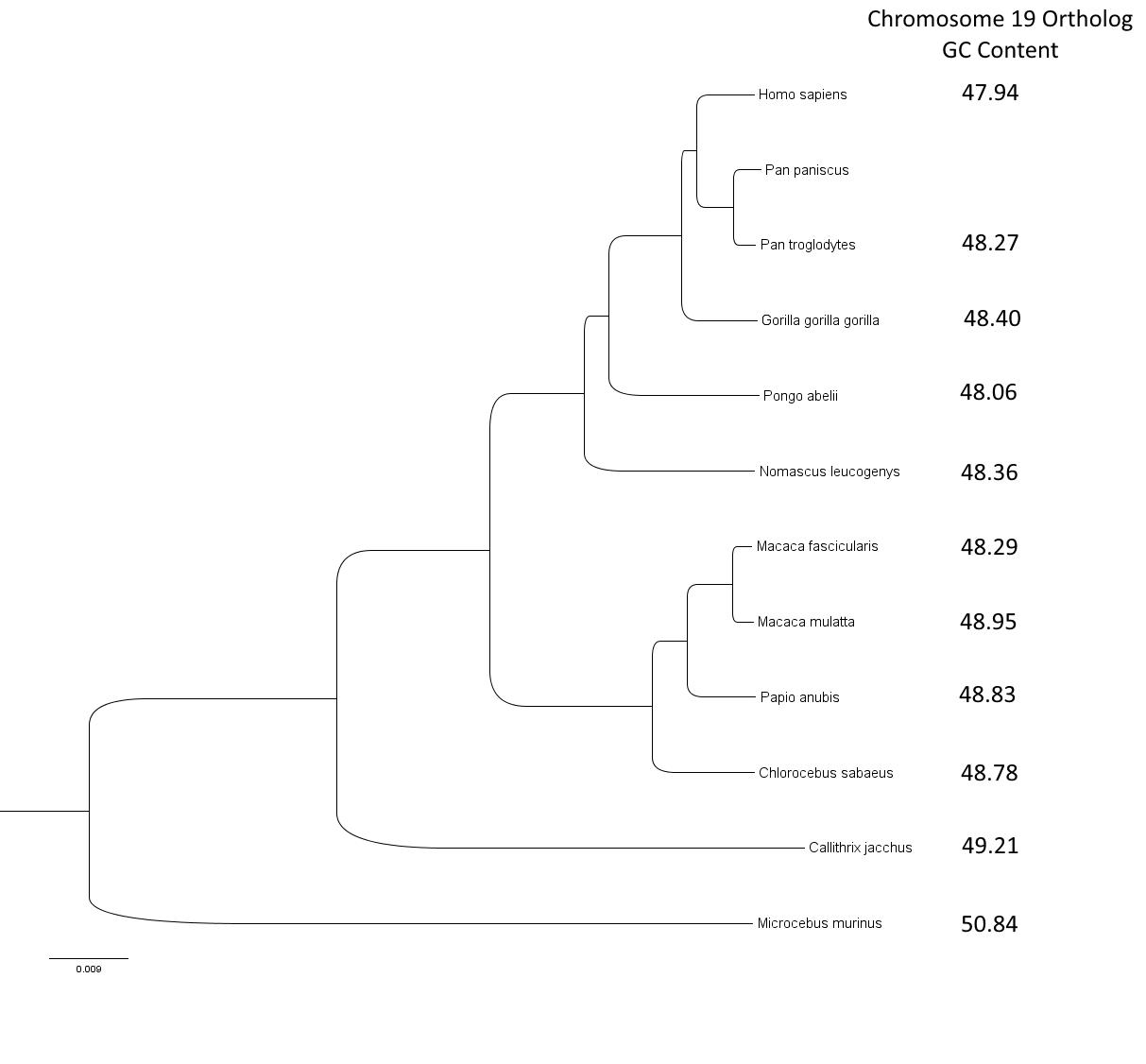


Fig S1.

Nonhuman primate phylogenetic tree showing the chromosome 19 ortholog GC content for species in the tree. The tree topology is based on the species tree used in the Ensembl Compara pipelines (<https://ensembl.org/info/genome/compara/species_trees.html>).

**A.**


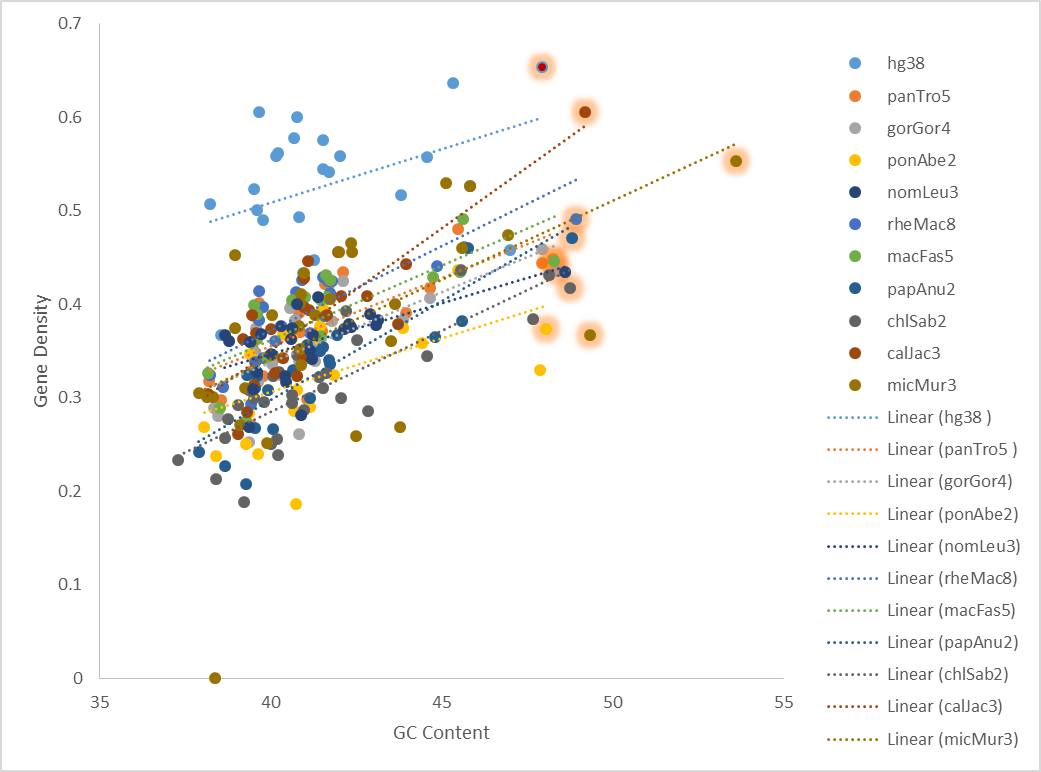


**B.**

| **Assembly** | **r_s_** | **two-tailed p value** |
| --- | --- | --- |
| hg38 | 0.3494 | 0.102002 |
| panTro5 | 0.7913 | 0.000004 |
| gorGor4 | 0.713 | 0.000092 |
| ponAbe2 | 0.6327 | 0.000912 |
| nomLeu3 | 0.641 | 0.00042 |
| rheMac8 | 0.8636 | < 0.000001 |
| macFas5 | 0.887 | < 0.000001 |
| papAnu2 | 0.8918 | < 0.000001 |
| chlSab2 | 0.7673 | 0.000001 |
| calJac3 | 0.8034 | 0.000004 |
| micMur3 | 0.5878 | 0.000318 |

Fig S2.
GC content compared to gene density in 100kbp windows of human and non-human primate chromosomes. A) Scatterplot of GC content compared to gene density by chromosome. Chromosome 19 orthologs are highlighted in red. B) Spearman's correlation coefficients and p values for GC content compared to gene density.
